# A meteorological dataset of the West African monsoon during the 2016 DACCIWA campaign

**DOI:** 10.1038/s41597-022-01277-7

**Published:** 2022-04-14

**Authors:** Martin Kohler, Geoffrey Bessardon, Barbara Brooks, Norbert Kalthoff, Fabienne Lohou, Bianca Adler, Oluwagbemiga Olawale Jegede, Barbara Altstädter, Leonard Kofitse Amekudzi, Jeffrey Nii Armah Aryee, Winifred Ayinpogbilla Atiah, Muritala Ayoola, Karmen Babić, Konrad Bärfuss, Yannick Bezombes, Guillaume Bret, Pierre-Etienne Brilouet, Fred Cayle-Aethelhard, Sylvester Danuor, Claire Delon, Solene Derrien, Cheikh Dione, Pierre Durand, Kwabena Fosu-Amankwah, Omar Gabella, James Groves, Jan Handwerker, Corinne Jambert, Norbert Kunka, Astrid Lampert, Jérémy Leclercq, Marie Lothon, Patrice Medina, Arnaud Miere, Falk Pätzold, Xabier Pedruzo-Bagazgoitia, Irene Reinares Martínez, Steven Sharpe, Victoria Smith, Andreas Wieser

**Affiliations:** 1 Institute of Meteorology and Climate Research, Karlsruhe Institute of Technology (KIT), Karlsruhe, Germany; 2 School of Earth and Environment, University of Leeds, Leeds, United Kingdom; 3 National Centre for Atmospheric Science, Leeds, United Kingdom; 4 Laboratoire d’Aérologie, Université de Toulouse, CNRS, UPS, Toulouse, France; 5 Department of Physics & Engineering Physics, Obafemi Awolowo University, Ile-Ife, Nigeria; 6 Institute of Flight Guidance, Technische Universität Braunschweig, Braunschweig, Germany; 7 Department of Physics, Kwame Nkrumah University of Science and Technology, Kumasi, Ghana; 8 Institute for Data Processing and Electronics, Karlsruhe Institute of Technology (KIT), Karlsruhe, Germany; 9 Meteorology and Air Quality Group, Wageningen University and Research, Wageningen, The Netherlands; 10 Sedoo, Observatoire Midi-Pyrénées, IRD, Toulouse, France; 11 Present Address: CIRES, University of Colorado, Boulder, CO USA; 12 Present Address: NOAA Physical Sciences Laboratory, Boulder, CO USA; 13 Present Address: Centre National de Recherche en Météorologie, Météo-France/CNRS, Toulouse, France; 14 Present Address: African Centre of Meteorological Applications for Development, Niamey, Niger; 15 Present Address: C.K. Tedam University of Technology and Applied Sciences, Navrongo, Ghana; 16 Present Address: Laboratoire Univers et Particules de Montpellier (LUPM), Université de Montpellier, CNRS/IN2P3, Montpellier, France; 17 Present Address: Observatoire Midi-Pyrénées, Toulouse, France; 18 Present Address: Laboratoire de l’Atmosphère et des Cyclones, UMR8105, CNRS, Université de La Réunion, Météo-France, Saint-Denis, La Réunion, France

**Keywords:** Atmospheric dynamics, Atmospheric chemistry

## Abstract

As part of the Dynamics-Aerosol-Chemistry-Cloud Interactions in West Africa (DACCIWA) project, extensive *in-situ* measurements of the southern West African atmospheric boundary layer (ABL) have been performed at three supersites Kumasi (Ghana), Savè (Benin) and Ile-Ife (Nigeria) during the 2016 monsoon period (June and July). The measurements were designed to provide data for advancing our understanding of the relevant processes governing the formation, persistence and dissolution of nocturnal low-level stratus clouds and their influence on the daytime ABL in southern West Africa. An extensive low-level cloud deck often forms during the night and persists long into the following day strongly influencing the ABL diurnal cycle. Although the clouds are of a high significance for the regional climate, the dearth of observations in this region has hindered process understanding. Here, an overview of the measurements ranging from near-surface observations, cloud characteristics, aerosol and precipitation to the dynamics and thermodynamics in the ABL and above, and data processing is given. So-far achieved scientific findings, based on the dataset analyses, are briefly overviewed.

## Abstract (summary)

**Table:** 

Measurement(s)	Wind speed, Wind direction, Temperature, Relative humidity, Soil Moisture, Soil Temperature, Air Pressure, Radiative Fluxes near the surface • range, elevation azimut, radial velocity, wind vectors • Brightness temperatures • Reflectivity, range, elevation azimut • Trace gas concentrations • Aeorosol optical depth • Sky images, visible and infrared • Temperature, Humidity and Windprofiles • Temperature, Relative Humidity and Black Carbon Concentration • Temperature, Humidity, vertical wind speed
Technology Type(s)	weather station, energy balance station • Lidar, Sodar, UHF-Profiler • Microwave Radiometer • Rain radar and Cloud radar • Trace gas analyser • Photometer • Cloud camera • Radiosoundings • UAV • Eddy Correlation
Factor Type(s)	Precipitation and Cloudformation • turbulent fluxes of heat and moisture • Vertical temperature, humidity and wind profiles
Sample Characteristic - Environment	Atmosphere
Sample Characteristic - Location	West Africa

## Background & Summary

Before DACCIWA, the skill of numerical weather and global climate models concerning the forecast of clouds and precipitation was overall low at the immediate Guinea Coast and southern West Africa during the summer monsoon^1,2^, possibly due to representation problems of the so-called Gulf of Guinea maritime inflow phenomenon^3,4^. Low clouds tended to be underestimated in many of these models, leading to too much solar incoming radiation at the Earth’s surface^4^. The small number of observational investigations available showed that during the monsoon season, nights are characterised by the frequent formation of nocturnal low-level stratus clouds (LLSCs), with typical cloud-base heights of a few hundred metres above ground. They can last the whole morning before being eroded by daytime ABL mixing^5,6^. According to a satellite-based study^7^, their extent covers an area of around 800 000 km^2^ and, thus, exerts a considerable effect on the regional climate. However, the understanding of the mechanisms and processes driving the West African atmospheric boundary layer (ABL) clouds diurnal cycle has been hindered by the dearth of high-quality observations. Only a few model studies were performed to investigate the mechanisms for cloud formation, persistence and dissolution^8,9^. Therefore, a more realistic representation of clouds and convection in southern West Africa in weather forecast and regional climate models was aimed at.

A number of processes expected to be relevant for the formation of LLSCs in this region have been listed by Adler *et al*.^9^ and Kalthoff *et al*.^10^ including large-scale advection, orographic lifting, lifting related to gravity waves, latent heat release, and vertical mixing of moisture due to shear-generated turbulence below the nocturnal low-level jet (LLJ). With respect to the latter process, shear-induced turbulence in the upper part of the LLJ and in the lower part of the African Easterly Jet (AEJ) could, depending on the position of the AEJ, mix the wet monsoon flow and the dry Saharan air layer above. Prior to DACCIWA it was not clear to what extent the nocturnal LLSC formation, their morning dissolution and the growth of the cloud-topped daytime ABL on the following day depend on free tropospheric moisture content and middle and upper-level clouds^10,11^. Aerosols emitted from the main metropolitan areas on the Gulf of Guinea coastline (Abidjan, Accra and, Lagos) and advected by the south-westerly monsoon flow also influence LLSC macro-physical characteristics in southern West Africa by affecting cloud microphysical properties such as cloud droplet and ice crystal formation and size-distribution^3,12^. The relevance of the different processes and their spatial variation were neither fully understood nor verified by observations, thus the need for further observation and analysis.

**Fig. 1:**
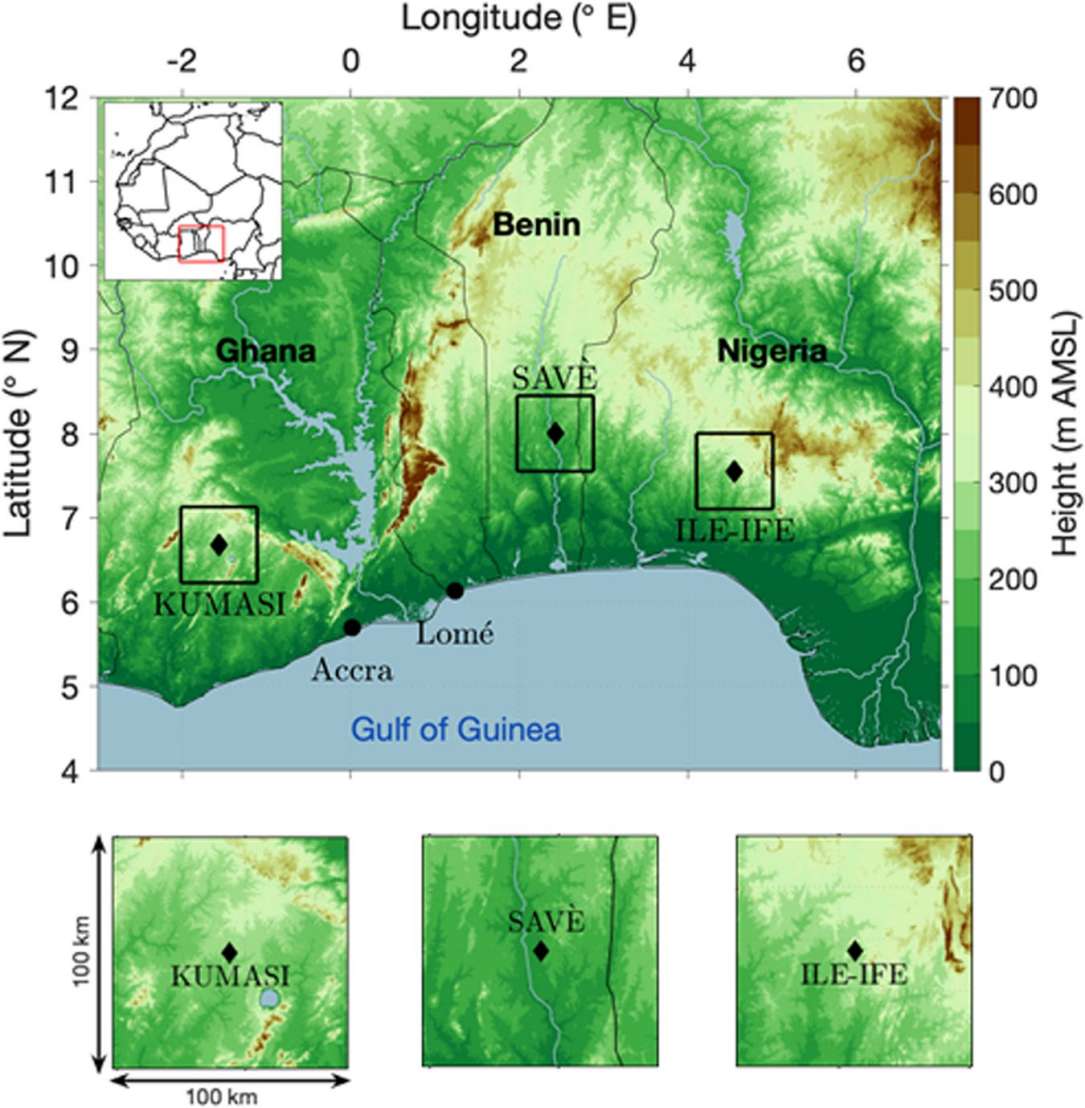
Location of the DACCIWA investigation area (red box) in West Africa and positions of the three supersites Kumasi in Ghana, Savè in Benin, and Ile-Ife in Nigeria (black diamonds). Zoomed-in orography in the 100 km per 100 km centred on the three supersites Kumasi, Savè, and Ile-Ife (lower panels). Thin solid lines indicate country borders with country names (adapted from Kalthoff *et al*.^10^).

The overall goal of the Dynamics-Aerosol-Chemistry-Cloud Interactions in West Africa (DACCIWA) project (European Union Framework Programme (FP7/2007–2013) under grant agreement no. 603502) was to significantly improve monitoring capacities and the scientific understanding of key interactions between surface-based emissions, atmospheric dynamics and chemistry, clouds, aerosols, and the climate of West Africa and to implement these findings into the next generation of weather and climate models^3^. Therefore, from mid-June to end of July 2016, as part of DACCIWA, an extensive measurement/observational campaign was conducted in the region: one of the objectives being to obtain high-quality observations that would allow the identification of the relevant processes impacting the formation of LLSC and the ABL diurnal cycle. The ground-based measurement strategy was designed such that the relevant processes expected to be involved in LLSC formation were recorded. To achieve this, measurements at three supersites: Kumasi (Ghana), Savè (Benin), and Ile-Ife (Nigeria) (Fig. 1) were complemented by additional radiosoundings from existing national and reactivated African Monsoon Multidisciplinary Analysis (AMMA) networks^13^ and flights with three research aircraft^14^. A detailed description of the field activities during summer 2016 is given by Flamant *et al*.^14^. Knippertz *et al*.^15^ overview the synoptic conditions that prevailed during the period of the DACCIWA campaign and Kalthoff *et al*.^10^ describe the meteorological conditions at the three ground-based supersites and the characteristics of the LLSC with associated features. This paper provides a detailed description of the measurements at the ground-based supersites and the available data and demonstrates that the collected dataset allowed identifying and describing the relevant processes for the LLSC formation and the development of the ABL diurnal cycle.

## Methods

### Experimental design, measurement strategy and data acquisition

The DACCIWA ground-based measurement campaign lasted from 14 June to 30 July 2016 and encompassed the three weeks of the airborne campaign (27 June to 16 July 2016), during which three European aircraft conducted research flights across Ivory Coast, Ghana, Togo and Benin^14^. During the ground-based campaign, a comprehensive set of instruments was deployed at the three DACCIWA supersites (Fig. 1), namely near the cities of Kumasi, Savè, and Ile-Ife, in zones where the formation of LLSC has been identified as frequent^8^. The clouds develop between the Guinean coast and 10 ° N. So, the three sites at around 8° N filled up the sparse radiosonde monitoring network between the West African coast and the inland and lay on the South-North transects from Lomé–Savè and Lomé–Accra–Kumasi of the research aircraft flight patterns^14^. Furthermore, three sites at nearly the same latitude allowed to monitor the cloud-layer homogeneity for similar large-scale forcing associated to the maritime inflow phenomenon. The equipment was distributed to allow providing the main spatiotemporal characteristics of the LLSC (e.g., onset, duration, dissolution, cloud base height, sub-cloud conditions). The three stations are characterised by more (Kumasi, Ile-Ife) or less (Savè) hilly terrain with hills not higher than 300 m above station height. At each supersite, *in situ* and remote sensing observations of (i) near-surface meteorological parameters, soil temperature, soil moisture and chemicals, (ii) dynamics and thermodynamics in the ABL and above, (iii) cloud characteristics, aerosol and precipitation, and, (iv) surface energy balance components were performed (Supplementary Tables 1–4).

During the ground-based campaign, all instruments worked continuously, except the remotely piloted aeroplane systems (RPASs), radio- and tethered sondes. Daily radiosondes were released in Kumasi at 0600 UTC, while in Savè the sondes were launched at 0500 UTC in order to be synchronous with the launchings at operational radiosonde stations.

Daily online meetings between the three super sites were held to discuss the execution of an intensive observation period (IOP). Prerequisite for an IOP was the absence of mesoscale convective systems and favourable conditions for LLSC evolution in the investigation area. The weather prediction was based on numerical forecast models and decision for an IOP was made in coordination with the DACCIWA Operations Center (DOC; http://dacciwa.sedoo.fr/) located in Lomé, Togo^14^ during the period of the airborne campaign.

**Fig. 2:**
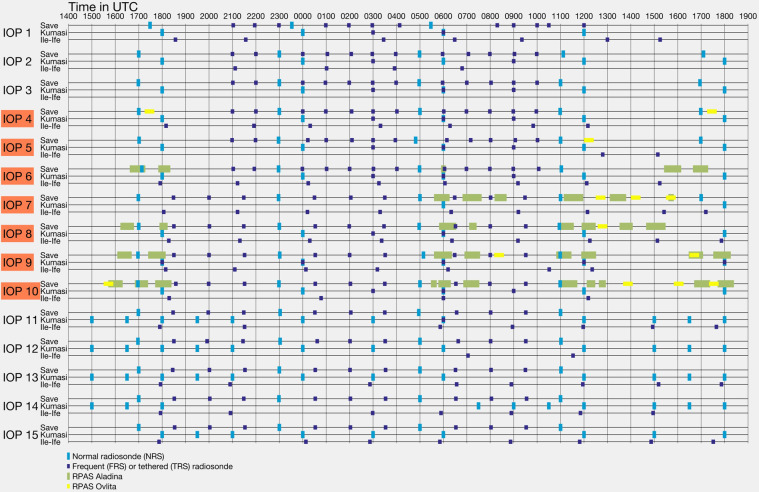
Timetable of complementary measurement during IOPs at the three super sites. IOPs with research aircraft flights are indicated in red.

During IOPs, additional measurements consisting of intensified radiosonde releases and flights from RPASs were performed. In total, 15 IOPs were conducted during the ground-based campaign: 7 of them during the aircraft campaign (Table 1). The IOPs (the day of the IOP is denoted as day D) ran for a 24-hour period commencing in the late afternoon on day D minus one (D-1). The launching times of the sondes were selected to sufficiently resolve the temporal development and spatial distribution of the LLSCs^10^. A detailed overview of the radiosonde and RPAS schedule for the three supersites is given in Fig. 2 and information on the instrumentation are available in Supplementary Table 1. The schedules for the three supersites look like:

- At Ile-Ife, tethered radiosondes (TRS) reaching heights of up to 900 m above ground level (AGL) were launched during IOPs, typically between 1800 UTC on day D-1 and 1800 UTC on day D. Intervals were approximately 3-hourly or 6-hourly, depending on availability of helium for the balloon platform and the operational condition of the electric winch. IOPs 3, 5, and 12 were only partially completed due to technical issues (shortage of helium).
- At Kumasi, there were 4 normal radiosondes (NRS, i.e. reaching up to the tropopause) with 6-hour intervals, the first one was launched at 1800 UTC on D-1 and the last one at 1200 UTC on D. In addition, frequent radiosondes (FRS) were launched up to an altitude of 650 m AGL at 0300 UTC, 0600 UTC and 0900 UTC. At 0600 UTC, 2 FRS were launched simultaneously with NRS to evaluate the performances of the FRS^16^. An excess of radiosondes led to the decision to increase the number of NRS toward the end of the campaign. For IOP 10 to 15, technical issues in the FRS ground station reduced the number of FRS.
- At Savè, there were 5 NRSs with 6-hour intervals, the first one was released at 1700 UTC on D-1 and the last one at 1700 UTC on D. Due to lack of radiosondes, the 1700 UTC radiosoundings on D were cancelled at Savè in the course of the campaign (after IOP 7). FRS up to an altitude of around 1500 m AGL were launched in between the NRSs. During IOPs 1–6, FRS were launched hourly between 2100 UTC on D-1 and 1000 UTC on D. Starting at IOP 7, FRS were launched in 90-minute intervals between 1830 UTC on D-1 and 0930 UTC on D in order to better capture the evening transition phase. RPAS OVLI-TA performed profile flights in the afternoon during periods when no FRS was launched. RPAS ALADINA performed flight pattern consisting of vertical profiles and horizontal legs during periods when stratus was present in the morning, when stratus was breaking and in the evening during the transition to stable conditions.

**Table 1:** IOP dates in 2016.

IOP #	Date	Aircraft flights	IOP #	Date	Aircraft flights
1	17/06	No	9	10/07	Yes
	18/06			11/07	
2	20/06	No	10	13/07	Yes
	21/06			14/07	
3	25/06	No	11	16/07	No
	26/06			17/07	
4	28/06	Yes	12	20/07	No
	29/06			21/07	
5	30/06	Yes	13	23/07	No
	01/07			24/07	
6	02/07	Yes	14	26/07	No
	03/07			27/07	
7	04/07	Yes	15	29/07	No
	05/07			30/07	
8	07/07	Yes			
	08/07

### Near-surface measurements

In Ile-Ife, an energy balance station was set up over a closely-cropped grass surface at the measurement site. The configuration of the used eddy-covariance (EC) system^17^ comprised of a sonic anemometer and a Lambda Instruments Corporation (LI-COR) infrared gas analyser both installed on a 2.1 m mast. Co-located with the EC system was a four-component net radiometer placed on a 1.7 m radiation stand and a soil-heat flux plate buried at a depth of −0.02 m and a soil moisture probe at − 0.05 m. A 15 m tower positioned nearby provided every 10 s multi-level measurements of wind speed at 5 selected heights (1.14, 1.88, 3.3, 6.3 and 12.1 m AGL), arranged in a log-linear fashion. With the exception of the 1.88 m AGL level, measurements of air temperature and relative humidity were provided with all the tower data averaged every minute. The EC system sampled every 0.1 s to produce the turbulent temperature, moisture and wind components. The integrated datasets were further reduced to 30-min averages to determine sensible and latent heat fluxes, among other meteorological parameters.

In Kumasi, the energy balance parameters were measured with a LI-COR gas analyser co-located at 3.5 m AGL with a sonic anemometer sampling respectively CO_2_ concentrations, wind speed and direction and sonic temperature every 0.05 s and stored as 60 s average in the dataset. Before averaging, the CO_2_ concentration, wind speed and direction have been used to provide an estimation of the turbulent fluxes every 30 min. A radiation sensor at 1.68 m AGL was sampling shortwave and longwave radiation every 5 s. Additionally, 6 soil moisture, soil temperature and soil heat flux probes, buried at a depth of −0.08 m, sampled soil heat flux, soil temperature and soil water potential every 5 min. In addition to the energy balance tower, an automatic weather station has been set up at 2 m AGL measuring air temperature, relative humidity, pressure, wind speed, wind direction, and precipitation with one-minute resolution.

In Savè, two towers were deployed on two different vegetation covers. The first one was deployed over a mix of grass and bushes^10^, with the instrumentation from the observation system KITcube^18^. This tower was equipped with sensors sampling every 1 s air temperature, relative humidity (2 m AGL), air pressure (1.5 m AGL), surface temperature and soil heat flux (−0.05 m). Longwave and shortwave radiation was measured with the same sampling rate at 3 m AGL. Data from this tower have been aggregated every minute and every ten minutes. Soil temperature and soil moisture (−0.05, −0.10, −0.30, −0.50 m) were measured every 10 min. In addition, a sonic anemometer deployed at 4 m AGL was sampling wind direction, wind speed and sonic temperature every 0.05 s and a LI-COR gas analyser sampled humidity with the same sampling rate. EC method has been used to calculate 30-min turbulent fluxes^19^. The second tower, deployed over a mixed corn field provided the surface energy balance terms and additional chemistry measurements through chemicals probe measuring respectively O_3_, NO, NO_2_, NH_3_, and CO every 10 s. O_3_, NO, and NO_2_ probes were deployed at 8.1 m AGL, CO probes at 5 m AGL and NO and NH_3_ probes have been deployed at 0.2 m AGL. A weather station (7.7 m AGL) recorded every 60 s precipitation and every 0.1 s air temperature, relative humidity and air pressure. The data from this device have been then aggregated over 60 s. Every 0.1 s water content (average between −0.01 and −0.31 m), soil temperature (average between −0.065 and −0.0165 m) and soil heat flux (−0.05 m) were recorded and then aggregated over 60 s. Precipitation (1.3 m AGL), incoming and outgoing long and shortwave radiations (7.16 m AGL) were sampled every 60 s. In addition, a sonic anemometer and a LI-COR gas analyser sampled the three wind components, the sonic temperature, the air humidity and the CO_2_ concentration every 0.1 s in order to estimate 30-min turbulent fluxes. Furthermore, biogenic soil fluxes of NO and NH_3_ were measured at the Savè site with the use of a chemiluminescence analyser, and the closed dynamic chamber technique^20^. Fluxes are given as daily averaged fluxes, calculated from 8 to 25 fluxes measured each day.

### Measurements of dynamics and thermodynamics in the boundary layer and above

#### Dynamical parameters

At all three sites, sodars (SOnic Detection and Ranging) were deployed to measure horizontal wind profiles with a vertical resolution of 20 m in Ile-Ife, 10 m at Kumasi and Savè, in the lowest 510 m AGL in Ile-Ife, 1000 m AGL in Kumasi, 600 m AGL in Savè and averaged at a temporal resolution of 10 min at Ile-Ife and Kumasi and 30 min at Savè.

In Savè, two wind lidars^18^ were deployed: the wind lidar Windcube was operated in vertical stare mode to provide vertical wind speed profiles with around 6 s temporal and 20 m vertical resolution from 40 to 600 m AGL. The wind lidar Windtracer performed a scan pattern, which combined vertical stare mode with range-height indicator (RHI) and plan-position indicator (PPI) scans. Every 30 min, two PPI scans at 15° and 75° elevation angles and four RHI scans (two along and two across the mean wind direction) were conducted taking about 5 min in total. The remaining 25 min, the lidar was looking vertically. During vertical stare mode, vertical wind speed profiles were obtained from around 400 to 5000 m AGL, thus extending the range of the Windcube, with 1 s temporal and 40 m spatial resolution. From RHI and PPI scans, the horizontal wind speed profiles were derived by the velocity–azimuth display (VAD) method and information on the spatial structure of the radial wind field between 400 and 9000 m distance, with 75 m spatial resolution along the laser beam, was obtained. Additionally, an ultra-high- frequency (UHF) wind profiler^21^ was deployed in Savè to measure horizontal wind profiles every 2 min. It was used in two modes^22^: a low acquisition mode covering the lowest part of the atmosphere (0–3500 m AGL) with a vertical resolution of 75 m to get a detailed profile of the horizontal wind in the boundary layer; a high acquisition mode covering a larger part of the atmosphere (0–4000 m AGL) with a vertical resolution of 150 m to obtain more information about the general horizontal wind profile.

#### Thermodynamic parameters

At Kumasi and Savè, microwave radiometers^23^ were installed providing integrated water vapor (IWV), liquid water path (LWP) as well as temperature and humidity profiles. While the radiometer at Kumasi performed a combination of vertical stare mode and boundary layer scans, the radiometer at Savè was able to perform additional (PPI) scans providing information on the spatial distribution of IWV and LWP every 30 min.

#### *In situ* measured dynamical and thermodynamic parameters

In Ile-Ife, TRS reaching heights of up to 900 m AGL were providing temperature and relative humidity profiles. In Kumasi and Savè, a combination of NRS reaching heights up to 20 km AGL, and FRS reaching heights to 650 m and 1500 m AGL, in the respective sites, provided temperature, relative humidity, wind speed and wind direction profiles.

Two RPASs have been flying over Savè. The RPAS OVLI-TA is a 3 kg airplane equipped with a 5-hole probe (in ongoing development) and temperature, humidity and pressure sensors. Only slow measurements were validated at the time of the campaign. OVLI-TA was flying vertical profiles most of the time below 600 m AGL in spirals of about 100 m radius. It reached 1000 m AGL maximum. A total of 20 flights were performed, each one with about 20 min duration. The RPAS ALADINA^24,25^ was operated for a total flight duration of 53 h at the local airfield in Savè, 4 km north-east of the supersite. The payload consists of meteorological sensors with a fast temporal resolution for measuring turbulent parameters and a micro aethalometer in order to observe black carbon mass concentrations. Vertical profiles were performed up to the maximum possible height of 1500 m AGL, i.e. below cloud base. In addition, horizontal flights were operated for studying turbulent fluxes at small-scale along 2 km long horizontal paths perpendicular to the prevailing wind direction.

### Cloud characteristics, aerosol and precipitation

At Ile-Ife and Savè, sun photometers^26^ recorded aerosol optical depths (AOD). While the sun photometer in Ile-Ife was hand-held to measure daily AOD values, the automatic sun photometer in Savè provided 30 min AOD values and precipitable water. Infrared radiometers were providing information on the cloud base temperature: at Ile-Ife, a hand-held infrared radiometer was operated every 3 hours measuring cloud-base temperature, at Savè 2 infrared radiometers as part of the microwave radiometer continuously measured brightness temperature at two frequencies, which represents cloud-base temperature in the presence of clouds.

At Kumasi and Savè, micro rain radars (MRRs^27^) were measuring rain rate, liquid water content and drop size distribution every 60 s. The vertical coverage at Kumasi was up to 1085 m AGL at range intervals of 35 m, and up to 6000 m AGL at range intervals of 200 m at Savè. Ceilometers recorded backscatter every 10 s up to 10 000 m AGL at range intervals of 5 m at Kumasi, while in Savè the vertical coverage is up to 15 000 m AGL at range intervals of 15 m and the temporal resolution in the file is 60 s.

Cameras have been deployed at 2 m AGL at Kumasi and 7.4 m AGL at Savè, to capture visible (daytime) and infrared (night-time) image of the sky every second in Kumasi and every 2 minutes in Savè.

In Savè, a cloud radar^18^ was operated combining vertical stare mode and RHI scans. In vertical stare mode, it provided radial velocity and reflectivity profiles from 150 m AGL to 15000 m AGL (depending on the backscatter concentration) at a vertical resolution of 30 m and a temporal resolution of 10 s. Every 30 minutes, two RHI scans in the prevailing wind direction and two RHI scans perpendicular to this direction were performed at 45°- elevation angle. The X-band rain radar performed a volume scan every 5 minutes using 11 elevation angles between 0.7° and 60° followed by a vertical stare scan to record reflectivity, radial velocity, differential reflectivity, specific differential phase, and further parameters of the precipitation distribution at a maximum range of 100 km. A Joss-Waldvogel distrometer^28^ measured near-surface drop size distribution every 60 s.

## Data Records

The present dataset with associated metadata is available on the Base Afrique de l’Ouest Beyond AMMA Base (BAOBAB) database http://baobab.sedoo.fr/DACCIWA/ where it is organised by country (Benin, Ghana, Nigeria), instrument and measured parameter. In total, 70 data records were generated and 7 DOIs have been created. All the data recorded in Ile-Ife can be found in Oluwagbeniga *et al*.^29^ and in Kumasi in Brooks *et al*.^30^. The data recorded at Savè site are structured as follows: all the data recorded by UPS are stored in Derrien *et al*.^20^. The data sets from KIT include near-surface measurements^31^, cloud and precipitation measurements^32^ and thermodynamic data from surface-based remote sensing^33^. The meteorological and black carbon data measured with the unmanned research aircraft ALADINA of the TU Braunschweig are available in Bärfuss *et al*.^34^.

The data formatting strategy has been designed with the aim to make direct comparisons between sites. All data recorded except the cloud cameras at Kumasi and Savè are available in NetCDF format, Savè X-band radar data are additionally packed as tar.gz. The cloud camera in Kumasi provides images in AVI format, the images in Savè are stored in JPG format and are archived as tar.gz. Similar aggregation times have been used for fluxes estimates. The supplementary tables are splitting the dataset into 3 subjects i.e., measurements of dynamics and thermodynamics in the boundary layer and above (Supplementary Table 1), measurements of cloud characteristics, aerosol and precipitation (Supplementary Table 2), near surface measurements (Supplementary Table 3), and energy balance measurements (Supplementary Table 4).

## Technical Validation

Before and during the campaign, it was necessary to calibrate the individual sensors and measurement systems and to perform other quality controls to ensure the data quality of the DACCIWA data sets. Consequently, the standard meteorological sensors (e.g. temperature, humidity, air pressure, wind speed) were calibrated prior to the campaign. For example, for temperature and humidity this was done either in certified facilities (climate chambers) or at the manufacturer’s premises. Anemometers were calibrated in certified wind tunnels or have been calibrated by the manufacturer. The equipment from the energy balance stations, like radiation instrumentation, was subject to regular calibrations by the manufacturers. The trace gas analysers operated at Savè supersite were calibrated regularly with reference gases. This procedure is described in detail by Pacifico *et al*.^35^. Concerning the radiosoundings, we relied on the manufacturer’s calibrated sondes (in warranty) (Kumasi, Ile-Ife) or even performed ground calibrations prior to releases of the sondes (Savè). The RPAS OVLI-TA and ALADINA used manufacturer calibrated sensors and ALADINA additionally calibrated the system’s sensor according to Bärfuss *et al*.^36^ Concerning remote sensing systems: The microwave radiometers were calibrated on site with liquid nitrogen (according to the calibration instructions of the manufacturers). Standard retrievals from the manufacturer were used (Kumasi) or the retrievals were trained on about 13000 soundings from Abidjan (Savè). The sun photometers in use were either calibrated by the manufacturer (Ile-Ife) or in accordance with the AERONET procedures^26^ prior to the campaign (Savè). All other remote sensing systems like wind lidars, cloud and rain radars, wind profiler and sodars were undergone regular maintenance procedures by the manufacturer before shipping them to the campaign in West Africa.

**Fig. 3:**
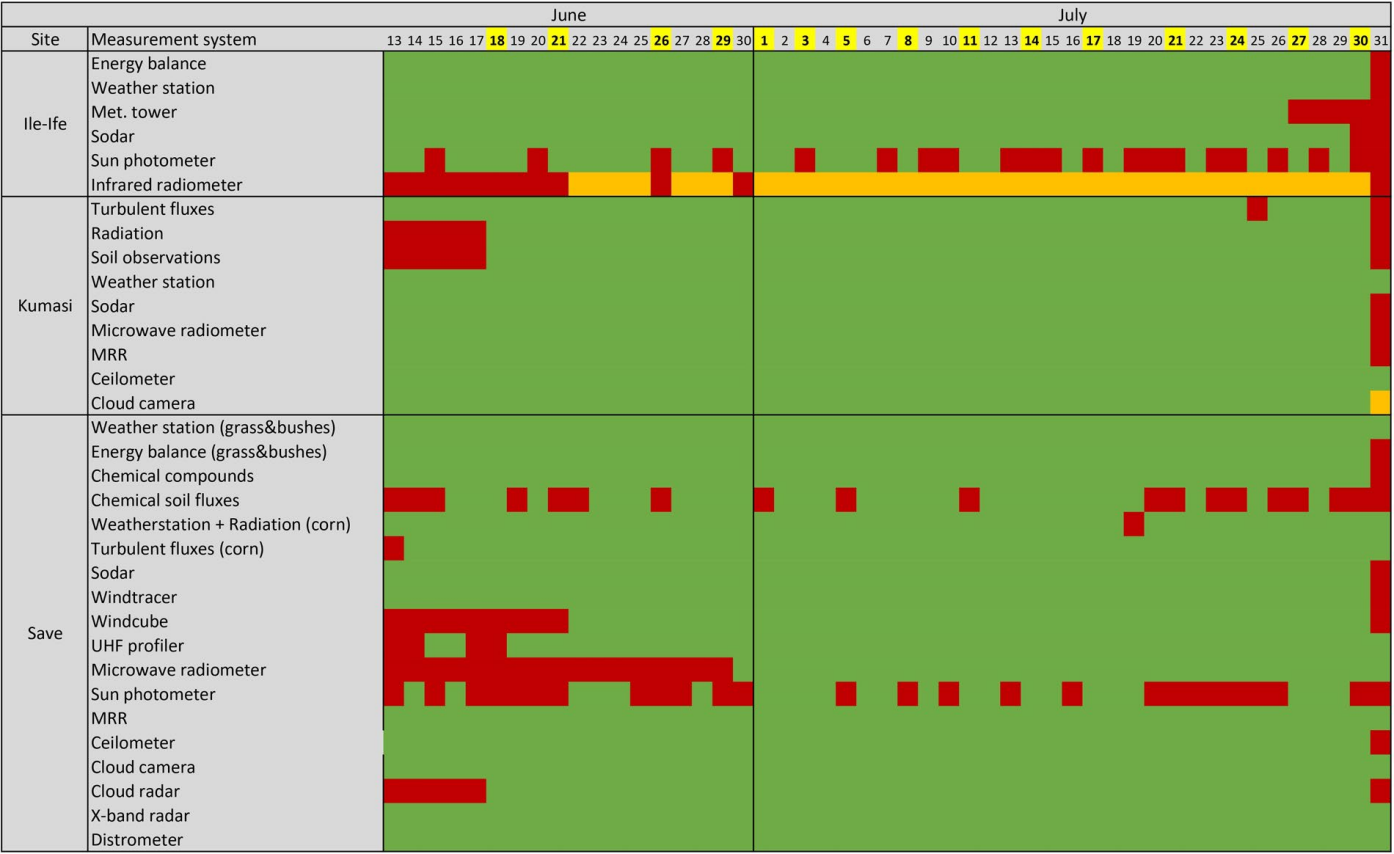
Data availability of the different measurement systems at the three supersites on the individual days. Green: data complete, orange: data partly available, red: data missing. The IOPs (day D) are indicated in yellow.

**Fig. 4:**
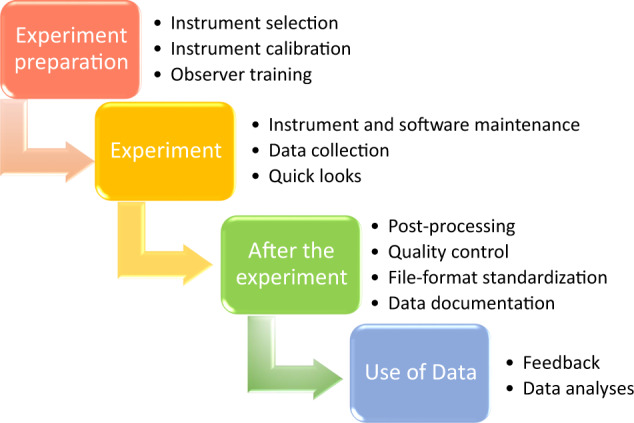
Data quality control in DACCIWA experiment preparation, data collection, processing, archiving and sharing.

As most of the meteorological parameters were measured simultaneously by different sensors, this redundancy allows cross-checking of the observations. By constant monitoring and regular maintenance of the instruments we tried to keep the loss of data low and the data quality high. Maintenance of the instruments and sensor failures (instrument, time, action taken) were protocoled in logbooks. Figure 3 shows an overview of the data availability after quality control. An overview of the whole series of quality control measures that were undertaken and which were implemented are summarised accordingly (Fig. 4):

1. Experiment preparation period: Observation sites, observation instrumentation and operating specifications were formulated and instruments were selected according to the operating specifications and scientific aims. To ensure the accuracy of the observation, instruments calibrations were performed before sending the instruments to the sites, then during the deployment instruments were aligned. To ensure the consistency of observations, an observer training was formed.
2. Experiment: a daily maintenance routine consisting of technical inspections, instrumentation cleaning, software maintenance, instrument self-calibration (when available) and data back-up has been set up and an experimental logbook filled. This routine ensures that instrument alignment and calibration is maintained through the campaign as well as any anomalies or exceptional events recorded and any accidental loss of data prevented. Data collection and quick looks were performed to check the quality and the integrity of the data through the campaign (http://dacciwa.sedoo.fr).
3. After the experiment: each institution applied their own post-processing method to the instrumentation that belongs to them and agreements between institutions were made on the file format and some derived parameter products. Quality-controlled data including meta data were published on the BAOBAB repository.
4. Use of data: user feedback was considered and the technical and scientific quality of the dataset was proved in numerous scientific publications which successfully investigated the processes relevant for the diurnal cycle of the ABL and the nocturnal LLSC evolution.

Kalthoff *et al*.^10^ show that extended LLSCs form frequently during the night-time and persist into the following day in the investigation area in June and July, while their characteristics (formation and dissolution time, cloud base height) vary considerably from day to day. These results apply for individual supersites and also between them. Dione *et al*.^37^ analysed the occurrence and characteristics of the monsoon flow, the LLJ and the maritime inflow at Savè supersite. They found that the maritime inflow and the LLJ are regularly present in the area, arriving e.g. at Savè between 1800 and 1900 UTC. Differential horizontal cold air advection, associated with the maritime inflow, and LLJ-generated turbulence cause the erosion of the stable nocturnal surface inversion. The associated cooling in the nocturnal ABL is crucial to reach saturation^38^. Quantification of the heat budget terms show that on average 50% of the cooling prior to the LLSC formation is due to horizontal cold air advection, 20% by radiative flux divergence and about 22% by sensible heat flux divergence in the presence of the LLJ. Detailed heat budget analysis of an individual night shows that the horizontal cold-air advection could even contribute up to 70% to cooling^39^. Advection of specific humidity by the maritime inflow is of minor relevance for the formation of LLSCs. The comparison of nights with and without LLSCs reveals that three major mechanisms are crucial whether LLSCs form or not: the onset time and strength of the LLJ, the horizontal cold air advection and the background moisture level^40^.

The individual studies allowed Lohou *et al*.^41^ to develop a conceptual model of the diurnal cycle of the LLSCs in southern West Africa. The cycle can be divided into four main phases: during the first two phases, denoted as the stable and the jet phase, the relative humidity increases until the air is saturated and the LLSCs form. The humidity increase is due to cooling of the air, mainly caused by horizontal cold air advection (50%). The remaining half is mainly due to divergence of net radiation and sensible heat flux. The third phase is the stratus phase, which starts when the LCCs have formed in the night and lasts until the onset of surface-buoyancy-driven turbulence on the following day. During the stratus phase, a mixing of the sub-cloud layer by shear-driven turbulence below the nocturnal LLJ core and the radiative cooling at the LLSC top influence the height of the cloud base and the coupling of the cloud to the Earth’s surface^42^. The fourth phase (convection phase) comprises the breakup of the LLSC due to convection. The breakup time, which depends partially on LLSC-surface coupling, has a considerable impact on the energy balance of the surface and, consequently, on the convective ABL depth, which was found to vary by a factor of 2 from day-to-day^41,42^. The observational data were also used to initialise large eddy simulations (LES) to perform sensitivity experiments investigating the impact of different processes (radiation, entrainment, turbulent fluxes and subsidence) on the diurnal stratocumulus-to-cumulus transition in the morning^43^. It turns out that the radiation term, which is the main source term of LLSCs liquid water content (LWC) during the night, decreases progressively during the day with the increase of solar radiation. After sunrise, the entrainment, enhanced by the rising of the cloud deck, is the leading factor for the decrease of LWC. The stronger the wind shear the stronger the role of the entrainment term. This result makes sense when considering the wind shear above the nocturnal LLJ core.

Beside the investigation of the processes governing the diurnal cycle of the LLSCs, the data from the supersites were used for additional studies. Haslett *et al*.^44^ used the radiosoundings to understand high aerosol optical depth values present across the region. They found evidence that these are driven by the high moisture level and are then moderated by changes in aerosol abundance. Altstädter *et al*.^45^ could show that the observed black carbon layer measured at the Savè super site was transported from the south with maritime inflow and was mixed vertically downwards when the nocturnal LLJ has formed. Performing high-resolution chemistry-transport modelling in combination with the surface and radiosonde data, Deroubaix *et al*.^46^ could characterise the relative contributions of different sources (i.e., emissions from several large coastal cities) to the air quality at Savè super site. Deetz *et al*.^47^ used the observations from the Savè and Kumasi super sites in their model simulations to investigate the aerosol radiative effects and their impact on clouds and atmospheric dynamics. The soil fluxes of nitric oxide (NO) and ammonia (NH_3_) observed over four different land cover types at Savè were successfully used for a model comparison with the Goddard Earth Observing System Chemistry Model (GEOS-Chem)^35^. Reinares Martines *et al*.^48^ analysed a warm-rain episode over southern West Africa using X-band radar measurements from Savè in combination with LES. The radiosoundings at Kumasi supersite were also applied to assess the quality of a newly developed reusable radiosonde systems^16^.

The numerous studies highlight that the datasets collected at the three supersites allowed to successfully solve several of DACCIWA questions and is a valuable addition to already available datasets like those from the African Monsoon Multidisciplinary Analysis (AMMA)^49,50^ project and the West African Science Service Centre on Climate Change and Adapted Land Use (WASCAL)^51,52^ project. Further exploitation of the data e.g. could use the wealth and quality of surface energy balance, *in-situ* and remote sensing profiling data to investigate the mean and turbulence characteristics of the cloud-topped convective boundary layer. Up to now, detailed studies are mainly based on Savè measurements. Even if the Kumasi and Ile-Ife sites were less instrumented, some of the cloud cycle characteristics at these two sites could be analysed and compared to the results obtained for Savè. This would provide a broader view on the ABL clouds. Furthermore, the spatial distribution of convective precipitation in dependence on convective instability could be investigated using rain radar observation.

## Supplementary information

Supplementary tables

## Data Availability

Here, the most important information about the used software is given. More information is included in the Supplementary Tables 1 to 4. For Ile-Ife supersite, the proprietary software EasyFlux DL v. 2.3 provided by Campbell Scientific for acquisition and analysis of the turbulent fluxes data is used (https://www.campbellsci.com/easyflux-dl). This software is written and supplied along with CSAT3/LI-7500 system. For Kumasi supersite, all codes produced to quality control and produce the data are stored under https://github.com/barbarabrooks/DACCIWA–Matlab. For example, the Matlab programme calculating the turbulent fluxes is stored under DACCIWA–Matlab/FL1/NC/NC flux estimates/write_NC_variables_FLUX_EST_v2.m. The other codes concern the automatic weather stations, Ceilometer, MRR, radiometer, rapid sondes, sodar, and radiosondes. At the Savè supersite, the turbulent fluxes of the KIT energy balance station were calculated with the TK3.11 software^19,53^ available under https://zenodo.org/record/20349. For the UPS energy balance station, the turbulent fluxes have been estimated with the EC method EddyPro® Software (Version 6.2.0) from LI-COR Environmental, available under https://www.licor.com/env/support/EddyPro/topics/whats-new.html. The vertical profiles of the wind deduced from the UHF wind profiler were calculated with a software developed in Laboratoire d’Aérologie^54^. This software has been used to process many previous campaign datasets^21,22,54–56^. The atmospheric quantities of the microwave radiometer at Savè were obtained with a retrieval algorithm provided by the University of Cologne^23,57–59^ available under ftp://gop.meteo.uni-koeln.de/pub/loehnert/mwr_data_flow/. The algorithm was trained on about 13000 radiosonde profiles measured at Abidjan, Ivory Coast, between 1980 and 2014. The cloud base height from ceilometer backscatter was retrieved applying the manufacturer’s algorithm (Manual - Lufft - CHM 15k. https://www.lufft.com/products/cloud-height-snow-depth-sensors-288/ceilometer-chm-15k-nimbus-2300/, 2017). Cloud radar uses IDL software provided by Metek^60^.
