# Supplementary material for: A meteorological dataset of the West African monsoon during the 2016 DACCIWA campaign
Source: Sci Data. 2022 Apr 14;9:174. doi: 10.1038/s41597-022-01277-7 (PMC9010450; doi:10.1038/s41597-022-01277-7)
Supplement: Supplementary file 1 — Supplementary tables [file 41597_2022_1277_MOESM1_ESM.pdf]

Table of contents

Page	
1	Table 1: Measurements of dynamics and thermodynamics in the boundary layer and above
2	Table 2: Measurements of cloud characteristics, aerosol and precipitation
3	Table 3: Near-surface measurements
4	Table 4: Energy balance measurements

Table 1: Measurements of dynamics and thermodynamics in the boundary layer and above

Site	Provider	Sensor annotation	Instrument provider/ manufacturer	Type	Measured parameter	Derived parameter	Derivation method	Vertical resolution in m/ vertical coverage in m AGL	Observation strategy/ time resolution/ time resolution in file	DOI	Data repository	Filename
Ile-Ife	OAU	Sodar	Metek	PCS 2000	u, v	ws, wd	Sodar2000_V3.1.2.16	20/ 30-510	continuous/ 600 s/ 600 s	10.6096/ DACCIWA. 1702	OAU_SODAR_Ile-Ife	Ile_Ife_OAU_Sodar_YYYYMMDD.nc
		Tethered radiosonde	Graw	GS-E, DFM09	T, RH	P, td, sh, D, theta, Tv	Grawmet 5.09.03.13	1/ 0-600	discontinous, every 3 hrs during IOP (between 1800 UTC on D and 1700 UTC on D+1)/ 1 s/ 1 s		OAU_ TetheredRadiosonde_ Ile-Ife	Ile_Ife_OAU_Tethered_Radiosonde_YYYYMMDD_21.nc
Kumasi	NCAS	Sodar	Scintec AG	MFAS	u, v, w, bsc	ws, wd, stdu, stdv, stdw, Qflags(wv, wd, u, v, w, bsc)	FASRun v2.19	10/ 0-970	continuous/ 600 s/ 600 s	10.6096/ DACCIWA. 1663	Sodar - Kumasi	ncas-sodar-2_kumasi_YYYYMMDD_backscatter-winds_v2.1.nc
		Microwave radiometer	Radiometer Physics	HATPRO-G4	BT at 14 frequencies in the microwave range , ele azi	Qflags(BT)	TPB_NN_RPG_v00101 - developed by RPG Radiometer Physics	height depend 10-300 / 0-10000	continuous/ 2 s/ 2 s		Scanning Microwave Radiometer : Brightness Temperature Profiles - Kumasi	ncas-scanning-radiometer-1_kumasi_YYYYMMDD_brightness-temperature_v1.2.nc
						TBL, Qflags (TBL)			continuous/ 2 s/ 600 s		Scanning Microwave Radiometer : Boundary Layer Temperature Profiles - Kumasi (BLT)	ncas-scanning-radiometer-1_kumasi_YYYYMMDD_boundary-layer-temperature-profile_v1.2.nc
						RH, sh, Qflags (RH, sh)			continuous / 2 s/ ~200 s		Scanning Microwave Radiometer : Stability Indicies-Kumasi	ncas-scanning-radiometer-1_kumasi_YYYYMMDD_moisture-profiles_v1.2.nc
						TT, Qflags(TT)			continuous/ 2 s/ ~200 s		Scanning Microwave Radiometer : Tropospheric Temperature Profiles- Kumasi (T)	ncas-scanning-radiometer-1_kumasi_YYYYMMDD_full-troposphere-temperature-profiles_v1.2.nc
						IWV, LWP, Qflags(IWV, LWP)		---	continuous/ 2 s/ 2 s		Scanning Microwave Radiometer : IWVLWP - Kumasi (IWV,LWP)	ncas-scanning-radiometer-1_kumasi_YYYYMMDD_iwv-lwp_v1.2.nc
						SI, Qflags(SI)		---	continuous/ 2 s/ ~200 s		Scanning Microwave Radiometer : Moisture Profiles Kumasi (AH)	ncas-scanning-radiometer-1_kumasi_YYYYMMDD_stability-indices_v1.2.nc
		Weather station	Davis Instruments	Vantage Pro 2 Wireless	T, RH, P, ws, wd, prec, RR , LWDR, UV-Index	SWDR, Qflags(T, RH, P, ws, wd, prec, RR, LWDR, SWDR)		---	continuous/ 2 s/ 2 s		Scanning Microwave Radiometer : Surface Met - Kumasi	ncas-scanning-radiometer-1_kumasi_YYYYMMDD_surface-met_v1.2.nc
		Normal radiosonde	Vaisala	DigiCora Sounding System with RS92SGP or RS41SG	T, RH, ws, wd, bs, P (RS92SGP)	P (RS41SG), Qflags (T, RH, ws, wd)	MW41	~4/ 0-20 000	discountinuous, every day during the campaign at 0600 UTC. Five radiosounding during IOP day every 6 hours (from 1800 UTC on day D to 1800 UTC on day D+1)/. 1 s/ 1 s		Radiosonde - Kumasi	ncas-radiosonde-2_kumasi_YYYYMMDD-hhmm_sonde_v1.2.nc
		Frequent radiosonde	Sparv Embedded	Windsond S1H2-R	T, RH, P, ws, wd	Qflags(T, RH, P, ws, wd)	Windsond Receiver Application 2.11	~8/ 0-650	discountinuous,every 3 hours during IOP from 0300 UTC on day D+1 to 0900 UTC on day D+1)/ 3 s/ 3s		Rapid Sonde - Kumasi	ncas-rapidsonde-2_knust_YYYYMMDD-hhmmss_sonde_v2.nc
Savè	KIT	Sodar	Scintec AG	MFAS	u ,v, bsc	ws, wd	APRun 1.45	10/ 30-600	continuous/ 1800 s/ 1800 s	10.6096/ DACCIWA. 1659	KIT_Sodar_Save	Save_KIT_SOD_YYYYMMDD.nc
		Wind lidar	Lockheed Martin	Wind Tracer HYB	ele, azi, bsc<br>ele, azi, rv<br>ele, azi, SNR		MiDAS Version 1.13.C.03	Vertical stare: 40/ 370-5470 Scanning Resolution along beam: 75/ Maximum range along beam 390 - 9900	continuous/ 500 Hz/ 1 (vertical stare), 0.1 s (scanning)		KIT_Doppler Lidar_ Save	Save_KIT_DOPPLERLIDAR_BS_YYYYMMDD.nc<br>Save_KIT_DOPPLERLIDAR_RV_YYYYMMDD.nc<br>Save_KIT_DOPPLERLIDAR_SNR_YYYYMMDD.nc
		Wind lidar	Leosphere	WINDCUBE8	w, CNR		WindSoft_LR - 1.1.9	20/ 40-600	continuous/ -/ 6.4 s		KIT_Doppler Lidar small_Save	Save_KIT_Windcube_YYYYMMDD.nc
		Microwave radiometer	Radiometer Physics	HATPRO-G4	BT at 14 frequencies in the microwave range BT at 2 frequencies in the infrared range, ele, azi	Qflags(BT)<br>HUA, Qflags(HUA)<br>TT, Qflags(TT)<br>TBL, Qflag(TBL<br>LWC, Qflags(LWP)<br>IWV, Qflag(IWV)	Software mwrpro-v04 provided by University of Cologne (Löhnert and Crewell 200320, Löhnert et al. 200921). Retrievals based on radiosonde profiles at Abidjan.	height depend 50-500/ 0-10000<br>---<br>---	continuous, combination of vertical stare and scanning mode/ -/ ~4 s TBL 900s/		KIT_HatproData - Save	Save_KIT_MWR_TB_YYYYMMDD.nc<br>Save_KIT_MWR_HUA_YYYYMMDD.nc<br>Save_KIT_MWR_TA_YYYYMMDD.nc<br>Save_KIT_MWR_TABL_YYYYMMDD.nc<br>Save_KIT_MWR_LWP_YYYYMMDD.nc<br>Save_KIT_MWR_IWV_YYYYMMDD.nc
	UPS	UHF wind profiler	Degreane Horizon	PCL1300	ASPT, SW, SKEW ,noise along each beam, rv	u, v, w, CN2	UPS/LA algorithm	High acquisition mode: 150 m/ 150-4000 m Low acquisition mode: 75 m/150-3500 m	continuous/ ~120 s/ ~120 s	10.6096/ DACCIWA. 1618	UPS_UHF WindProfiler Data_Save	dacciwa_uhf_high_mode_v1.nc<br>dacciwa_uhf_low_mode_v1.nc
		RPAS OVLITA	Campbell Scientific	SHT75	T, RH, GPS coordinates, IMU aircraft position, pressure differentials on radome, static pressure	theta, ws, wd, u, v,	UPS/LA algorithm	~3 m/ 0-1000 m Profiles were generally flown below 600 m	daytime discontinuous/ -/ 2 s		UPS-OVLI RPAS_Save	dacciwa_ovli_YYYYMMDD-hhmm_up_v01 .nc (ascent)<br>dacciwa_ovli_YYYYMMDD-hhmm_down_v01 .nc (descent)
		Normal radiosonde	Modem	M10 or M2K2DC	T, RH, GPS coordinates	td, ws, wd, u, v, P	Modem software	~5 m/ 0-20 000 m	discountinuous, every day during the campaign at 0600 UTC. Five radiosounding during IOP day every 3 h (from 1800 UTC on day D to 1200 UTC on day D+1)/ -/-		UPS_Radiosoundings_ Save	LAYYYYLAMMDDhhmm.nc
		Frequent radiosonde	Modem	M10	T, RH, GPS coordinates	td, ws, wd, u, v, P	Modem software	~5 m/ 0-1500 m	discountinous, 1 and 1.5 h on IOP day/ -/ -		UPS_Frequent Radiosoundings _ Save	LAYYYYMMDDhhmm.nc
	IFF	ALADINA	Vaisala<br>Innovative Sensor Technology<br>Amsys<br>TU Braunschweig<br>iMAR GmbH<br>EKO Instruments	HMP110<br>Rapid P14<br>TSYS01<br>FineWire and five-hole probe<br>imVRU<br>ML-01	slow T, slow RH<br>fast RH<br>slow T<br>fast T, P<br>WGS84 coordinates, speed vector in earth fixed coordinate system, pitch, roll, yaw,track angle, Mach number, true airspeed<br>SWDR, SWUR	theta, Rwet, D<br>ws, wd u, v, w, alpha, beta		variable/ 0-1500 m	discontinuous, vertical profile up to 1500 m AGL, cross pattern with straight legs of at least 1 km length at constant altitude with several altitudes per flight/ flights every in intervals of few hours during daytime / flight duration 40-50 min, resolution 0.01 s	10.6096/BAOBAB-DACCIWA.1701	IFF_ALADINA-RPAS_Save	id01_YYYYMMDD_record1_hhmm-hhmm_v2.nc

Abbreviations	Long name
alpha	Angle of attack
ASPT	Power_aspect_ratio
azi	Azimuth
beta	Angel, side slip
bs	Ballon ascent speed
bsc	Backscatter
BT	Brightness temperature
CN2	Structure coefficient of the air refravctive index
CNR	Carrier to noise ratio
D	Air density
D	Air density
ele	Elevation
HUA	Absolute hunidity
IMU	Inertial measurement unit
IWV	Integrated water vapour
LWC	Liquid water content
LWDR	Long-wave downwelling radiation
LWP	Liquid water path
P	Air pressure
prec	Total amount of precipitation
Qflags(XXX)	Quality flag of a specific parameter
RH	Relative humidity
RR	Rain rate
rv	wind, radial velocity or Doppler velocity
Rwet	Specific gas constant
sh	Specific humidity
SI	Atmospheric stability Indices
SKEW	vertical_velocity_skewness
SNR	Signal to noise ratio
stdX	Standard deviation of a specific parameter
SW	Vertical velocity doppler Spectral width
SWDR	Short-wave downwelling radiation
SWUR	Short-wave upwelling radiation
T	Air temperature
TBL	Temperature profile from boundary layer scan
td	Dew point temperature
thetha	Potential temperature
TT	Temperature, profile from full troposphere scan
Tv	Virtual temperature
u	Eastward wind component
v	Northward wind component
w	Vertical wind speed
wd	Wind direction
ws	Wind speed

Table 2: Measurements of cloud characteristics, aerosol and precipitation

Site	Provider	Sensor annotation	Instrument provider/ manufacturer	Type	Measured parameter	Derived parameter	Derivation method	Vertical resolution in m/ vertical coverage in m AGL	Observation strategy/ time resolution/ time resolution in file	DOI	Data repository	Filename
Ile-Ife	OAU	Hand-held infrared thermometer	Optron	IT330 274	CBT	CT		-	discontinuous/ 3 h/ 3 h	10.6096/ DACCIWA. 1702	OAU_CBT_Ile-Ife	oau-cloud-base-temp-june_july_2016.csv
		Photometer	TENUM	Calitoo Sun Photometer	T, P, raw465, raw540, raw619, ele	aot465, aot540, aot619		Calitoo v2.5	discontinuous/ < 10 s/ < 10 s		OAU_AOD_Ile-Ife	Ile_Ife_OAU_AOD_YYYYMMDD.nc
Kumasi	NCAS	Rain radar	Metek	MRR 2	Z	DSD, Qflags	Metek MRR Control 6.0.0.6	35/ 35-1085	continuous/ 60 s/ 60 s	10.6096/ DACCIWA. 1663	Concentration Profiles - Kumasi	ncas-mrr-1_kumasi_YYYYMMDD_size-concentration-spectra_v1.2.nc
					Z	RR, LWC, Zv , PIA	Metek MRR Control 6.0.0.6				Rain and Reflectivity Profiles - Kumasi	ncas-mrr-1_kumasi_YYYYMMDD_rain-lwc-velocity-reflectivity_v1.2.nc
		Ceilometer	Campbell Scientific	CS135	Aerosol bsc	-	-	5/ 10- 250	continuous/ 10 s/ 10 s		Ceilometer - Kumasi	ncas-ceil-1_kumasi_YYYYMMDD_backscatter_v1.2.nc
		Cloud camera	Ycam	Protect Outdoor Camera 1080	Visible and Infrared sky image	-	-	Measuring height : 2 m AGL	continuous/ 1 s/ 1 s		Camera - Kumasi	YYYY-MM-DD_hh.avi
Savè	KIT	Rain radar	Metek	MRR 2	Z	Zv, RR, LWC, MPp, Nd, PIA	proprietary software by Uli Blahak	200/ 200-6000	continuous/ 60 s/ 60 s	10.6096/ DACCIWA. 1686	KIT_MRR_Save	int01_YYMMDD.dat.gz
			Selex International	Meteor50 DX	Z, rv, sw, ZDR, uZ, KDP, PhiDP, rhoHV, uPhiDP, ele, azi		Rainbow 5.36.5	Horizontal radius of volume scan: 100 km	continous volume scan/ -/ 300 s		KIT_X-Band_Radar_Save	YYYYMMDD.tar.gz
		Cloud radar	Metek	MIRA36-S	VELg, Zg, LDR, RMSg, ele, azi	VELcl, Z	IDL software by METEK 33th conference on radar metorology, 2007	30/ 150-15000	continous, combination of vertical stare and scanning mode/ -/ 10 s (vertical stare), 1 s (scanning)		KIT_CloudRadar Data-Save	Save_KIT_CR_YYYYMMDD_v1.nc
		Distrometer	Ott	Joss-Waldvogel	DSD	Z, RR		Measuring height: 0.1 m	continous/ -/ 60 s		KIT_Distrometer_ Save	Save_KIT_JWD_YYYYMMDD.nc
		Ceilometer	Lufft	CHM15k Nimbus	beta_raw (normalized range corrected signal)	CBH, CD	Lufft 12.12.1 2.13 0.733	15/ 0-15000	continuous/ -/ 60 s		KIT_CeilometerData_Save	Save_KIT_CM_YYYYMMDD.nc
		Photometer	Cimel	CE316	only derived data available	AOD,PW, AP<br>RI<br>AOD_T, AOD_F, AOD_C<br>AOT absorption<br>AOT extinction<br>SSA<br>Af<br>Phase functions	AERONET Version 3 for AOD and Almucantar Retrievals Version 2, quality level 1.5	Sensor height: 1 m AGL	continuous/ 1800 s/ 1800 s	10.6096/ DACCIWA. 1659	KIT_SunPhotometer_ Save	SAVE_KIT_SUN_AODPWAP.nc<br>SAVE_KIT_SUN_SIZ.nc<br>SAVE_KIT_SUN_SDA.nc<br>SAVE_KIT_SUN_TAB.nc<br>SAVE_KIT_SUN_EOT.nc<br>SAVE_KIT_SUN_SSA.nc<br>SAVE_KIT_SUN_ASY.nc<br>SAVE_KIT_SUN_PFN.nc
	UPS	Cloud camera	Mobotix	S15	Visible and infrared sky image	images	-	Sensor height : 7.4 m AGL Horizontal full sky visible images 43 by 32 degree aperture for IR images	continuous/ 120 s/ 120s	10.6096/ DACCIWA. 1618	UPS_Cloud_camera - Savé	mobotix_gobe_inrab_YYMMDD.tar.gz
	IFF	RPAS ALADINA	Vaisala	HMP110	T, RH			Variable/ 0-1500	discontinuous, vertical profile up to 1500 m AGL, cross pattern with straight legs of at least 1 km length at constant altitude with several altitudes per flight/ flights in intervals of few hours during daytime/ flight duration 40-50 min, resolution 1 s	10.6096/ BAOBAB-DACCIWA. 1701	IFF_ALADINA-RPAS_Save	id01_YYYYMMDD_record1_hhmm-hhmm_v2_additional.nc
			Microaeth	AE-51	BC	BC low pass filtered						

Abbreviations	Long name
Af	Asymmetry factor
AOD	Aerosol optical depth
AOD_C	AOD, coarse mode
AOD_F	AOD, fine mode
AOD_T	AOD, total
aotXXX	Aerosol optical thickness in a specific wave length range
AP	Angstrom exponent
azi	Azimuth angle
BC	Black carbon concentration
beta_raw	normalized range corrected signal
bsc	Backscatter, aerosol
CBH	Cloud base height
CBT	Cloud base temperature
CD	Cloud depth
CT	Cloud type
DSD	Precipitation, drop size distribution
ele	Elevation angle
KDP	Specific differential phase
LDR	Linear de-polarization ratio
LWC	Liquid water content
MPp	Marshall Palmer fit parameters
Nd	Total number of drops per cubic meter
P	Air pressure
PhiDP	Differential propagation phase (PhiDP)
PIA	Path integrated attenuation
PW	Precipitable water
Qflags	General quality flags
Qflags(XXX)	Quality flag of a specific parameter
rawXXX	Aerosol, raw data in a specific wave length range
RH	Relative humidity
rhoHV	Co-polar correlation coefficient
RI	Refractive index
RMSg	Peak width
RR	Rain rate
rv	Radial velocity
SSA	Single scattering albedo
sw	Spectral width
T	Air temperature
uPHiDP	Uncorrected differential propagation phase
uZ	Uncorrected reflectivity
VELcl	Velocity of cloud drops and ice particles
VELg	Velocity of all particles (global)
Z	Reflectivity
ZDR	Differential reflectivity
Zg	Equivalent radar reflectivity of all targets
Zv	Reflectivity weighted velocity

Table 3: Near-surface measurements

Site	Operator	Sensor annotation	Instrument provider/ manufacturer	Type	Measured parameter	Derived parameter	Measuring heights in m AGL	Observation strategy/ time resolution/ time resolution in file	DOI	Data repository	Filename
Ile-Ife	OAU	Pressure sensor	Campbell Scientific	CSAT3		P	2.1	continuous/ 0.1 s/ 1800 s	10.6096/ DACCIWA. 1702	OAU_Met-Obs_Ile-Ife	Ile_Ife_OAU_MetObs_YYYYMMDD.nc
		Temperature/ humidity probe	Campbell Scientific/ Vaisala	HMP 60	T, RH	sh, D	2.1				
		Anemometer	Vector Instruments	A101 ML	ws		1.71	continuous/ 10 s/ 1800 s			
		Windvane	Vector Instruments	W200P	wd		1.71				
		Soil temperature probe	Campbell Scientific	T108	ST		-0.02, -0.05, -0.10, -0.30, -0.50				
		Soil moisture	Campbell Scientific	CS616	SM		-0.05				
		Net radiometer	Campbell Scientific/Hukseflux	NR01		Tsur	1.65				
		Rain gauge	Texas Instruments	TE525	prec		0.2	continuous/ 1800 s/ 1800 s			
		Net radiometer	Scientific/Hukseflux	NR01	SWDR, SWUR, LWDR, LWUR	Tsur, Qnet	1.65	continous/ 10 s/ 60 s		OAU_EnergyBalance_1min_Ile_Ife	Ile_Ife_OAU_EB1min_YYYYMMDD.nc
		Soil heat flux plates	Hukseflux	HFP01	shf		-0.02				
		Soil moisture	Campbell Scientific	CS616							
		Soil temperature probe	Campbell Scientific	T108	ST		-0.02, -0.05, -0.10, -0.30, -0.50				
		Temperature/humidity probe	Campbell Scientific	HMP60	T, RH		1.14, 3.3, 6.3, 12.1	continuous/ 10 s/ 60 s		OAU_Profile_Ile-Ife	Ile_Ife_OAU_15m_tower_YYYYMMDD.nc
		Anemometer	Vector Instruments	A101 ML	ws		1.14, 1.88, 3.3, 6.3, 12.1				
Kumasi	NCAS	Soil heat flux plates	Hukseflux	HFP01SC (x6)	shf	Qflag(shf)	-0.08	continuous/ 300 s/ 300 s	10.6096/ DACCIWA. 1663	Energy Balance 1 : Soil - Kumasi<br>Energy Balance 2 : Soil - Kumasi	ncas-energy-balance-1_kumasi_YYYYMMDD_soil_v1.2.nc<br>ncas-energy-balance-2_kumasi_YYYYMMDD_soil_v1.2.nc
		Soil temperature probe	Campbell Scientific	107 (x6)	ST	Qflags(ST)					
		Soil moisture/Soil matric potential	Campbell Scientific	253 (x6)	swp	Qflags(wp)					
		Net radiometer	Hukseflux	NR01	SWDR, SWUR, LWDR, LWUR	Qflags	1.68	continuous/ 5 s/ 5 s		Energy Balance 1 : Radiation - Kumasi	ncas-energy-balance-1_kumasi_YYYYMMDD_radiation_v1.2.nc
		Weather station	Davis Instruments	Vantage Pro 2 Wireless	T,RH,P,ws,wd, prec, RR, LWDR, UV-Index	SWDR, Qflags	2	continuous/ 60 s/ 60 s		Automatic weather station - Kumasi	ncas-aws-1_kumasi_YYYYMMDD_surface-met_v1.2.nc
		Sonic anemometer	Metek	uSonic-3	u, v, w, Tsonic	ws, wd, wsgust, wdgust, stdu, stdv, stdw, stdTsonic, Qflags(u, v, w, Tsonic)	3.5	continuous/ 0.05 s/ 60 s		Sonic Anemometer - Kumasi	ncas-sonic-1_kumasi_YYYYMMDD_mean-winds_v1.2.nc
		Fast humidity probe	LI-COR	LI-7500	T, P, H_2O, CO_2.	sh, mr, stdT, stdP, stdH_2O, stdCO_2, stdsh, stdmr, Qflags(T, P, H_2O, CO_2)	3.5	continuous/ 0.05 s /60 s		Licor - Kumasi	ncas-licor-1_kumasi_YYYYMMDD_mean-co2-h2o_v2.nc
Savè	KIT	Temperature / humidity sensor	Vaisala	HMP35A	T, RH		2	continuous/ 1 s/ 600 s	10.6096/ DACCIWA. 1690	KIT_MetData_Save	Save_KIT_EB10min_YYYYMMDD.nc
		Pressure device	SETRA	Model 270	P		1.5				
		Sonic anemometer	Gill Instruments	Solent R1012	ws, wd		4	continuous/ 0.05 s/ 600 s			
		Soil heat flux plates	Hukseflux	HFP01SC_05	shf		-0.05	continuous/ 1 s/ 600 s			
		Rain gauge	Thies	HP	prec		1				
		Infrared thermometer	Heimann	KT15	Tsur		2				
		soil moisture /soil temperature probe	SMG, University of Karlsruhe	SISOMOP	SM, ST		-0.05, -0.1, -0.3, -0.5	continuous/ 600 s/ 600 s			
		Net pyrgeometer	Kipp & Zonen	CG3	LWDR, LWUR		3	continuous/ 1 s/ 600 s			
		Net pyranometer	Kipp & Zonen	CM14	SWDR, SWUR		3				
		Temperature / humidity sensor	Vaisala	HMP35A	T, RH		2	continuous/ 1 s/ 60 s		KIT_MetData 1min_Save	KIT_MetData_1min_Save_YYYYMMDD.nc
		Pressure device	SETRA	Model 270	P		1.5				
		Sonic anemometer	Gill Instruments	Solent R1012	ws, wd		4	continuous/ 0.05 s/ 60 s			
		Soil heat flux plates	Hukseflux	HFP01SC_05	shf		- 0.05	continuous/ 1 s/ 60 s			
		Rain gauge	Thies	HP	prec		1				
		Infrared thermometer	Heimann	KT15	Tsur		2				
		Net pyrgeometer	Kipp & Zonen	CG3	LWDR, LWUR	stdLWDR, stdLWUR	3	continuous/ 1 s/ 60 s		KIT_Radiation 1min_Save\V1	Save_KIT_EB1min_Radiation_YYYYMMDD.nc
		Net pyranometer	Kipp & Zonen	CM14	SWDR, SWUR	stdSWDR, stdSWUR	3				
	UPS	Net radiometer	Kipp & Zonen	CNR4	SWDR, SWUR, LWDR, LWUR		7.16	continuous/ 60 s/ 60 s	10.6096/ DACCIWA. 1618	UPS_MetData-radiation_1min_Save	tower1min_gobe_inrab_YYYYMMDD_000000_l2a_v01_dacciwa.nc
		Radiometer	Kipp & Zonen	PQS1	PAR		7.3	continuous/ 0.1 s/ 60 s			
		Soil temperature probe	Campbell Scientific	T107	ST		Average between -0.065 and -0.0165 m				
		Soil moisture probe	Campbell Scientific	CS616	SM		2 sensors : (a) on the mound, (b) in the furrow. Average between -0.01 and -0.31 m				
		Soil heat flux plates	Hukseflux	HFP01	shf		-0.05				
		Rain gauge	Environmental Measurement	ARG100	prec		1.3	continuous/ 60 s/ 60 s			
		Barometer	Druck	RPT410F	P		3.3				
		Weather station	Vaisala	WXT520	T, RH, P		7.7	continuous/ 0.1 s/ 60 s			
					prec			continuous/ 60 s/ 60 s			
		Trace gas analyser	Thermo Fisher Scientific	49i	O_3	Qflags(O_3)	8.1	continuous/ 10 s/ 60 s		UPS_Chemical Compounds Composition Save	UPS_CCC_O3_YYYYMMDD.nc
			ThermoEnvironment Instrument	42CTL	NO	Qflags(NO)	8.1				UPS_CCC_NO_YYYYMMDD.nc
					NO_2	Qflags(NO_2)					UPS_CCC_NO2_YYYYMMDD.nc
			ThermoEnvironment Instrument	48C	CO	Qflags(CO)	5				UPS_CCC_CO_YYYYMMDD.nc

Abbreviations	Long name
CO	Carbon monoxide concentration
CO_2	Carbon dioxide concentration
D	Air density
H_2O	Water vapour concentraction
LWDR	Long-wave downwelling radiation
LWUR	Long-wave upwelling, radiation
mr	Mixing ratio
NO	Nitrogen oxide concentration
NO_2	Nitrogen dioxide concentration
O_3	Ozone concentration
P	Air pressure
PAR	Photosynthetically active radiation
prec	Total amount of precipitation
Qflags	General quality flags
Qflags(XXX)	Quality flag of a specific parameter
Qnet	Net radiation
RH	Relative humidity
RR	Rain rate
sh	Specific humidity
shf	Soil heat flux
SM	Soil moisture humidity
ST	Soil temperature
stdX	Standard deviation of a specific parameter
SWDR	Short-wave downwelling radiation
swp	Soil water potential
SWUR	Short-wave upwelling radiation
T	Air temperature
Tsonic	Sonic temperature
Tsur	Surfac temperature
u	Eastward wind component
v	Northward wind component
w	Vertical wind speed
wd	Wind direction
wdgust	Wind direction of gusts
ws	Wind speed
wsgust	Wind speed of gusts

Table 4: Energy balance measurements

Site	Provider	Sensor annotation	Instrument provider/ manufacturer	Type	Measured parameter	Derived parameter	Derivation method	Measuring height in m AGL	Sampling rate/ temporal resolution	DOI	Data repository	Filename
Ile-Ife	OAU	Pressure sensor	Campbell Scientific	CSAT3		P		2.1	0.1 s/ 1800 s		OAU_EnergyBalance_Ile_Ife	Ile_Ife_OAU_EB30min_YYYYMMDD.nc
		Temperature/humidity probe	Campbell Scientific/ Vaisala	HMP60	T, RH	sh, D		2.1	10 s/ 1800 s			
		Soil heat flux plates	Hukseflux	HFP01	shf			-0.02, -0.05				
		Soil temperature probe	Campbell Scientific	T108	ST			-0.02, -0.05, -0.10, -0.30, -0.50				
		Net radiometer	Campbell Scientific/Hukseflux	NR01	SWDR, SWUR, LWDR, LWUR	Tsur, Qnet		1.65				
		EC-System	Campbell Scientific<br>LI-COR	CSAT3<br>LI-7500	Tsonic	ws, wd, H, E,u*,TKE, Rf, stdu, stdv, stdw, stdTsonic, covuv, covuw, covuTsonic, covvw, covvTsonic, covwTsonic	EasyFlux DL v. 2.3	2.1	0.1 s/ 1800 s			
Kumasi	NCAS	Sonic anemometer	Metek	uSonic-3	u, v, w, Tsonic	covwH_2O, covwTsonic, H, E, MF_u, MF_v, Bo, BuF, Qflags	Aubinet et al. (2012)[18]	3.5	0.05 s/ 1800 s	10.6096/ DACCIWA. 1663	Flux Estimates - Kumasi	ncas-flux-1_kumasi_YYYYMMDD_flux-estimates_v1.nc
		Fast humidity probe	LI-COR	LI-7500	T, P, CO_2, H_2O							
Save	KIT	Sonic anemometer	Gill Instruments	Solent R1012		Varu, Varv, Varw, VarH2o, VarTsonic, wd, ws, shf, Qnet, H, E, u*, D, Z/L, Qflags(H, E, u*)	Eddy-Covariance Software TK311<br>Mauder et al. (2013)[19]	4	0.05 s/ 1800 s	10.6096/ DACCIWA. 1690	KIT_EnergyBalance_Save	KIT_EnergyBalance_SAVE_YYYYMMDD.nc
		Fast humidity probe	LI-COR	LI-7500								
	UPS	Sonic anemometer	Campbell Scientific	CSAT3	Tsonic	Varu, Varv, Varw, VarH2O, VarTsonic, VarCO2, ws, wd, H, E, F(CO2), u*, D, TKE, MF, L, Qflags(H, E, CO2, H2O, MF), spikes(u, v, w, CO2, H2O)	EddyPro (Li-Cor) V6.20	7.77	0.1 s/ 1800 s	10.6096/ DACCIWA. 1618	UPS_EnergyFlux 30min_Save	tower10Hz_gobe_inrab_YYYYMMDD_000000_l3_v01_dacciwa.nc
		Fast humidity probe	Campbell Scientific/<br>LI-COR	LI-7500	CO_2, H_2O							
		Soil Chemical fluxes	Thermo Fisher Scientific	17i		F(NO), stdF(NO), F(NH_3), stdF(NH_3), Soil Type	Closed dynamic chamber technique	0.2	10 s/ daily		UPS_Biogenic Soil Fluxes_Save	UPS_BiogenicSoilFluxes_20180125.nc

Abbreviations	Long name
Bo	Bowen ratio
BuF	Buoyancy flux
CO_2	Carbon dioxide concentration
covXX	Covariance of specific parameters
D	Air density
E	Latent heat flux
F(CO2)	Carbon dioxide flux
F(NH3)	Ammonia flux
F(NO)	Nitrogen oxide flux
H	Sensible heat flux
H_2O	Water vapour concentration
L	Obukhov length
LWDR	Long-wave downwelling radiation
LWUR	Long-wave upwelling radiation
MF	Momentum flux
MF_u	Momentum flux, eastward
MF_v	Momentum flux northward
P	Air pressure
Qflags	General quality flags
Qflags(XXX)	Quality flags of specific parameters
Qnet	Net radiation
Rf	Richardson flux number
RH	Relative humidity
sh	Specific humidity
shf	Soil heat flux
spikes(XXX)	Spikes of a specific parameter
ST	Soil temperature
stdX	Standard deviation of a specific parameter
SWDR	Short-wave downwelling radiation
SWUR	Short-wave upwelling radiation
T	Air temperature
TKE	Turbulent kinetic energy
Tsonic	Sonic temperature
Tsur	Surface temperature
u	Eastward wind component
u*	friction velocity
v	Northward wind component
VarXX	Variance of specific parameter
w	Vertical wind speed
wd	Wind direction
ws	Wind speed
Z/L	Monin Obukhov stabiliy parameter
